# Deficient knowledge in adult Turner syndrome care as an incentive to found Turner centers in Germany

**DOI:** 10.1530/EC-19-0418

**Published:** 2019-10-18

**Authors:** Elin Kahlert, Martina Blaschke, Knut Brockmann, Clemens Freiberg, Onno E Janssen, Nikolaus Stahnke, Domenika Strik, Martin Merkel, Alexander Mann, Klaus-Peter Liesenkötter, Heide Siggelkow

**Affiliations:** 1 Clinic of Gastroenterology and Endocrinology, University Medical Center Goettingen, Goettingen, Germany; 2 Endokrinologikum Goettingen, Goettingen, Germany; 3 Interdisciplinary Pediatric Center for Children with Developmental Disabilities and Severe Chronic Disorders, University Medical Center Goettingen, Goettingen, Germany; 4 Endokrinologikum Hamburg, Hamburg, Germany; 5 Endokrinologikum Berlin, Berlin, Germany; 6 Endokrinologikum Hannover, Hannover, Germany; 7 Endokrinologikum Frankfurt, Frankfurt/Main, Germany

**Keywords:** Turner syndrome, adult height, cardiovascular involvement, medical care, Turner centers

## Abstract

**Objective:**

Turner syndrome (TS) is characterized by the complete or partial loss of the second sex chromosome and associated with a wide range of clinical manifestations. We aimed to assess the medical care of adult patients with TS in Germany.

**Design:**

Retrospective multicenter observational study.

**Methods:**

Data were collected from medical records of 258 women with TS treated between 2001 and 2017 in five non-university endocrinologic centers in Germany.

**Results:**

Mean age was 29.8 ± 11.6 years, mean height 152 ± 7.7 cm, and mean BMI 26.6 ± 6.3 kg/m^2^. The karyotype was known in 50% of patients. Information on cholesterol state, liver enzymes, and thyroid status was available in 81–98% of women with TS; autoimmune thyroiditis was diagnosed in 37%. Echocardiography was performed in 42% and cardiac MRI in 8.5%, resulting in a diagnosis of cardiovascular disorder in 28%. Data on growth hormone therapy were available for 40 patients (15%) and data concerning menarche in 157 patients (61%).

**Conclusion:**

In 258 women with TS, retrospective analysis of healthcare data indicated that medical management was focused on endocrine manifestations. Further significant clinical features including cardiovascular disease, renal malformation, liver involvement, autoimmune diseases, hearing loss, and osteoporosis were only marginally if at all considered. Based on this evaluation and in accordance with recent guidelines, we compiled a documentation form facilitating the transition from pediatric to adult care and further medical management of TS patients. The foundation of Turner Centers in March 2019 will improve the treatment of TS women in Germany.

## Introduction

With a prevalence of 1 in 2500 live female births and around 16,000 affected women in Germany, Turner syndrome (TS) is one of the most common chromosome aberrations. It is caused by the complete or partial absence of the second sex chromosome (1). The clinical appearance depends on the specific karyotype, which may include different mosaic forms. Main features of almost all the karyotypes are short stature and delayed or absent puberty (1, 2, 3). Most TS girls require estrogen replacement therapy to treat ovarian insufficiency and induce puberty. Although human growth hormone therapy (hGHT) has been established in TS patients, its efficacy varies and depends on many factors, including dose, duration, and age at treatment onset (4, 5, 6). Adult women in particular are at high risk of developing cardiac complications, especially aortic dilatations (7, 8), as well as metabolic syndrome including the onset and progression of obesity (9, 10). Furthermore, increased liver enzyme values, thyroid abnormalities, sensorineural hearing loss, and inflammatory bowel disease are relatively common in TS (3, 11, 12, 13, 14, 15). In addition, these women also display a tendency to suffer from low bone mineral density (16) and to develop autoimmune diseases (17). The overall life expectancy of women with TS is shortened by more than 10 years (18). Moreover, several studies have reported that the medical care of TS patients is unsatisfactory after transitioning from pediatric to adult care (2, 8, 19, 20, 21, 22, 23, 24). A number of reasons for this situation have been suggested. One aspect may well be a lack of adequate information for women with TS with respect to their health (21, 25). Some authors also attribute the insufficient care to a lack of the necessary personal responsibility resulting from the described impairment in social skills displayed by TS patients as a contributing factor (21, 25). Multidisciplinary centers capable of managing the care after childhood are important to improve and maintain the quality of medical care in women with Turner syndrome and were established in several countries during the last decade (26, 27, 28, 29, 30, 31, 32).

However, at the time of the study, there were no specialized centers for adult women with TS in Germany and general practitioners are not normally familiar with the complex comorbidities in TS. Mostly, after leaving pediatric care, these women are cared for by their primary care physician or gynecologist and are only sent to an endocrinologist for metabolic and thyroid control. In Germany, the majority of these girls are regularly seen by pediatric endocrinologists at university hospitals, non-university hospitals and in specialized private practices. However, they often do not receive adequate management of the transition to adulthood.

The aim of this study was to assess the nature of the medical care adult women with TS received in a number of non-university endocrinologic centers in Germany. These data are to be regarded as health service research documenting the care of adult women between the years 2001 to 2017. We were interested in the extent of the information documented by the attending endocrinologist. This retrospective data analysis was performed for women with TS in these endocrinologic centers prior to the publication of the clinical practice guidelines (33). These data contributed to the founding of the Turner-Syndrome Network in Germany in March 2019.

## Methods

### Patients

Data from 258 adult women with TS were analyzed retrospectively from August 2016 to June 2017. Inclusion criteria were a confirmed diagnosis of TS and a patient age of over 18 years (all participants were born prior to 1998). The women were adult patients at five different private, non-university endocrinologic centers in Germany between 2001 and 2017 (amedes group GmbH Goettingen, Berlin, Hamburg, Hannover and Frankfurt). These centers were not specialized in TS. The patients in this study were most frequently cared for by their primary care physician and sent once a year to one of these centers for metabolic and thyroid control. In some centers, the women had also been treated formerly as children in the pediatric clinic. However, we only evaluated the patients’ course through clinic as adults and not as children from previous clinical encounters.

Data concerning patients’ age (years), mass (kg), height (cm), BMI (kg/m^2^), karyotype, growth therapy, comorbidities, serum parameters, parents, and menarche were collected from the adult patients’ medical records.

### Ethics consideration

The study was approved by the Local Ethics Committee in Goettingen (18/2/07) and conformed to the Declaration of Helsinki, allowing the use of the anonymized data for research purposes. All patients were requested to give written informed consent to their individual data being used anonymously in the study. Patients who refused to give consent in the form of a signed statement were excluded from the study (*n* = 20).

### Laboratory parameters

Laboratory parameters were collected from the medical records retrospectively from the most recent time point of presentation (between 2001 and 2017). If different units for thyroid values were documented in the medical records in the different centers (in three centers it was pg/mL and in two pmol/L) the recorded values were converted to allow comparison (conversion factor: pmol to pg – factor: 0.651). All other values were documented in identical units. Laboratory parameters were initially analyzed only in one laboratory – however, as of 2014, three different laboratories were involved. Not all data were available for all patients, especially if treated according to specific issues or as a result of a change of residence.

Thyroid function was evaluated by measuring thyroid-stimulating hormone (TSH) (mU/L), free thyroid hormone (fT4, pg/mL) and free triiodothyronine (fT3, pg/mL). The antibodies (AB) TPO-AB/MAK-AB (thyroperoxidase-antibodies/microsomal auto antibodies) and TRAK-AB (thyrotropin-receptor auto antibodies) were categorized as positive or negative, according to the cut-off values as set by the analytical laboratories (TPO >35 U/mL, MAK >60 U/mL and TRAK >1.75 IE/L).

Liver enzymes included glutamate pyruvate transaminase (GPT) and glutamate oxaloacetate (GOT). The liver parameters were categorized as ‘increased liver enzymes’, ‘isolated increased γ-glutamyltransferases (gamma-GT)’, and ‘normal liver parameters’, according to the following cut-off values, where GPT >35 U/I, GOT >35 U/I, and gamma-GT >40 U/I were considered as increased.

We recorded the values of glycated hemoglobin (HbA1c) in %, low-density lipoprotein (LDL) in mg/dL and high-density lipoprotein (HDL) in mg/dL. Glucose metabolism was categorized as diabetes, insulin resistance, and normal metabolism, based on the diagnosis present in the medical records.

### Evaluation of the cardiac status

We used cardiac findings or the patients’ medical records to document the absence or presence of any heart involvement. As corresponding parameters, we noted congenital and acquired valve defects, aortic defects, and cardiac function. The date the last heart examination took place as well as imaging method, echocardiography or cardiac MRI, were documented.

### Statistics

We performed statistical analysis of the data with IBM SPSS Statistics 24 software, using the general linear model, multinomial logistic regression, and descriptive statistics. The significance level was set at *P* < 0.05. The general linear model was used to analyze metric parameters and multinomial logistic regression was implemented for nominal parameters. Statistical analyses were partially guided by Dr Gladitz Statistik-Service in Berlin, Germany.

## Results

We collected data from 258 patients with TS from five different specialist endocrinology centers in Germany. Baseline characteristics of the patients are depicted in Table 1. Mean patient age was 29.8 ± 11.6 years. Patient age at time of diagnosis was 12.1 ± 6.3 years, including all patients independent of hGHT (*n* = 258). The age at diagnosis of those who received hGHT was 7.6 ± 5 years (*n* = 58). In girls undergoing therapy, hGHT was started at 9.8 ± 3.4 years of age (*n* = 97; *n* = 41 age at diagnosis unknown); therefore, on average 2 years after diagnosis. Participants’ mean height was 152 ± 7.7 cm, which is about 14 cm less than the mean value for the female population in Germany (German Federal Office for Statistics 2017). The mean patient BMI was 26.6 ± 6.3 kg/m^2^. This BMI is 2.4 kg/m^2^ greater than the German average for the age band 25-30 years (German Federal Office for Statistics 2017). We found 47.4% of patients to be overweight (BMI >25 kg/m^2^) and 25% obese (BMI >30 kg/m^2^).

**Table 1:** Patient characteristics.

Parameter	Number of patients	Mean value	s.d.^a^	Normative data^b^	
Age (years)	258	29.8	11.6	25–30	30–35
Age at diagnosis (years)	152	12.1	6.3		
Height (cm)	252	152.8	7.7	167	167
Weight (kg)	241	62.1	15.3	65.4	67
BMI (kg/m^2^)	240	26.6	6.3	23.4	24.0

^a^s.d., standard deviation; ^b^German Federal Office for Statistics 2017.

**Figure 1:**
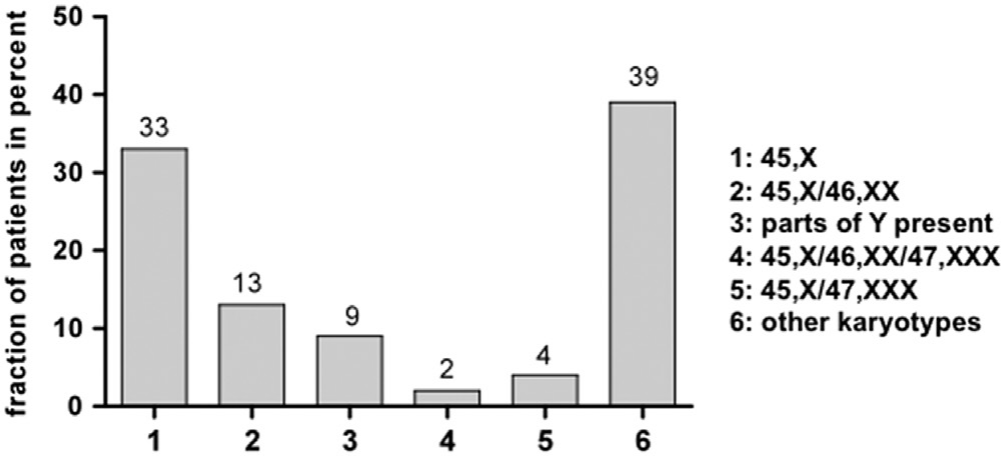
Distribution of karyotypes in patients with TS (documented in medical records).

The exact karyotype was documented in 50% of the patients. The karyotype 45,X was identified in 33%, mosaic forms (45,X/46,XX; 45,X/46,XX/47,XXX; 45,X/47,XXX) were found in nearly 20%. Parts of the Y chromosome were present in 9%. Other deletions and other mosaic forms were pooled in the group ‘other karyotypes’ (Fig. 1).

The medical information available during the history of medical care for the TS patients in our study is summarized in Table 2. Information concerning the cardiac status was documented in 43% of the participants. An MRI was performed only in 8.5% (*n* = 22). Almost complete information on the cholesterol state, liver enzymes, and thyroid status was available in 81–98% of women with TS (Table 2). Data concerning HbA1c were available in 74%, the mean HbA1c being 5.27 ± 0.54. According to documentation in the medical records, impaired glucose metabolism was diagnosed in 12% of women. The diagnosis of diabetes was assigned to 3.1% of patients with TS, insulin resistance to 8%. Information on the menarche (either spontaneous or induced) was found to be documented in 61% (*n* = 157 medical records). Menarche was spontaneous in approximately one-third of these women (*n* = 46, 29.3%). In the remaining patients (*n* = 111, 70.7%), menarche had to be induced. Mean age at time of menarche was 15.1 ± 2.2 years, corresponding information being available for 53.4% of all patients. Hormone replacement therapy (HRT) was administered to 81.4% of patients in total. Of these women 77.6% received treatment in oral form, 16.6% as dermal application, and in the remaining 5.8%, the type of application was not documented exactly. In this study, 8.5% of the patients were recorded as not having received HRT and no information was available for 10.1%.

**Table 2:** Medical information available for the selected parameters.

Parameter	Number of patients	%
Total	258	100
Cardiologic examination	112	43
Echocardiography	109	42
Cardiac MRI	22	8.5
TSH	255	98
LDL	210	81
HbA1C	192	74
Liver values	241	93
Menarche (spontaneous/induced)	159	62
Age at menarche	139	54
HRT	232	90
Type of HRT (oral/dermal)	198	77

HbA1C, glycated hemoglobin; HRT, hormone replacement therapy; LDL, low-density lipoprotein; MRI, magnetic resonance imaging; TSH, thyroid-stimulating hormone.

In the following, we further analyzed the selected comorbidities in more detail. An overview of the corresponding data relating to heart, thyroid metabolism, and liver values is depicted in Table 3. In our study, 31 patients (28% of the women examined) demonstrated cardiac involvement. Four women were diagnosed with stenosis of the aortic isthmus, six with ventricular septal defect (VSD) and five women were revealed to have a bicuspid aortic valve (Table 4). Cardiac monitoring involved echocardiography and cardiac MRI. Echocardiography was documented as having been performed in 102 patients and cardiac MRI in 22 patients (Table 2). Autoimmune thyroiditis was diagnosed in 37% of the patients at a mean age of 18 ± 9.4 years. Other thyroid diseases such as Graves’ disease, goiter, or autonomy of the thyroid were found in 3% of the women. Substitution with l-thyroxine was necessary in 99 patients (38%), with a mean dosage of 99.7 ± 38.4 µg/day (range 12.5–200 µg/day). Positive TPO-AB/MAK-ABs were found in 71 women (27%). TRAK-ABs were positive in 10 women (3.8%) in our study. Increased liver values were detected in 41% and isolated increased gamma-GT in 11% of the participants.

**Table 3:** Documented comorbidities in TS patients.

Comorbidity	Number (%) (total *n* = 258)	Number diagnosed	% of diagnosed	% of all patients
Cardiac involvement	112 (43%)			
With cardiac involvement^a^		31	28	12
Without cardiac involvement		81	72	31
Unknown involvement	146 (57%)			
Thyroid function	257 (100%)			
Autoimmune thyroiditis		95	37	37
Other thyroid diseases		8	3	3
No thyroiditis		154	60	60
Unknown involvement	1			
Hepatic function	241 (93%)			
Increased liver values		98	41	38
Increased gamma GT		26	11	10
Normal liver values		117	49	45
Unknown involvement	17 (7%)			

^a^Refer to Table 4 for details.

**Table 4:** Most frequently documented cardiac comorbidities in TS patients.

Documented cardiac comorbidities in TS patients (*n* = 31)	Number (%)
Stenosis of the aortic isthmus	4 (14%)
Bicuspid aortic valve	5 (17%)
Other heart diseases	10 (34%)
Combined heart defects	4 (14%)
VSD	6 (21%)

VSD, ventricular septum defect.

**Figure 2:**
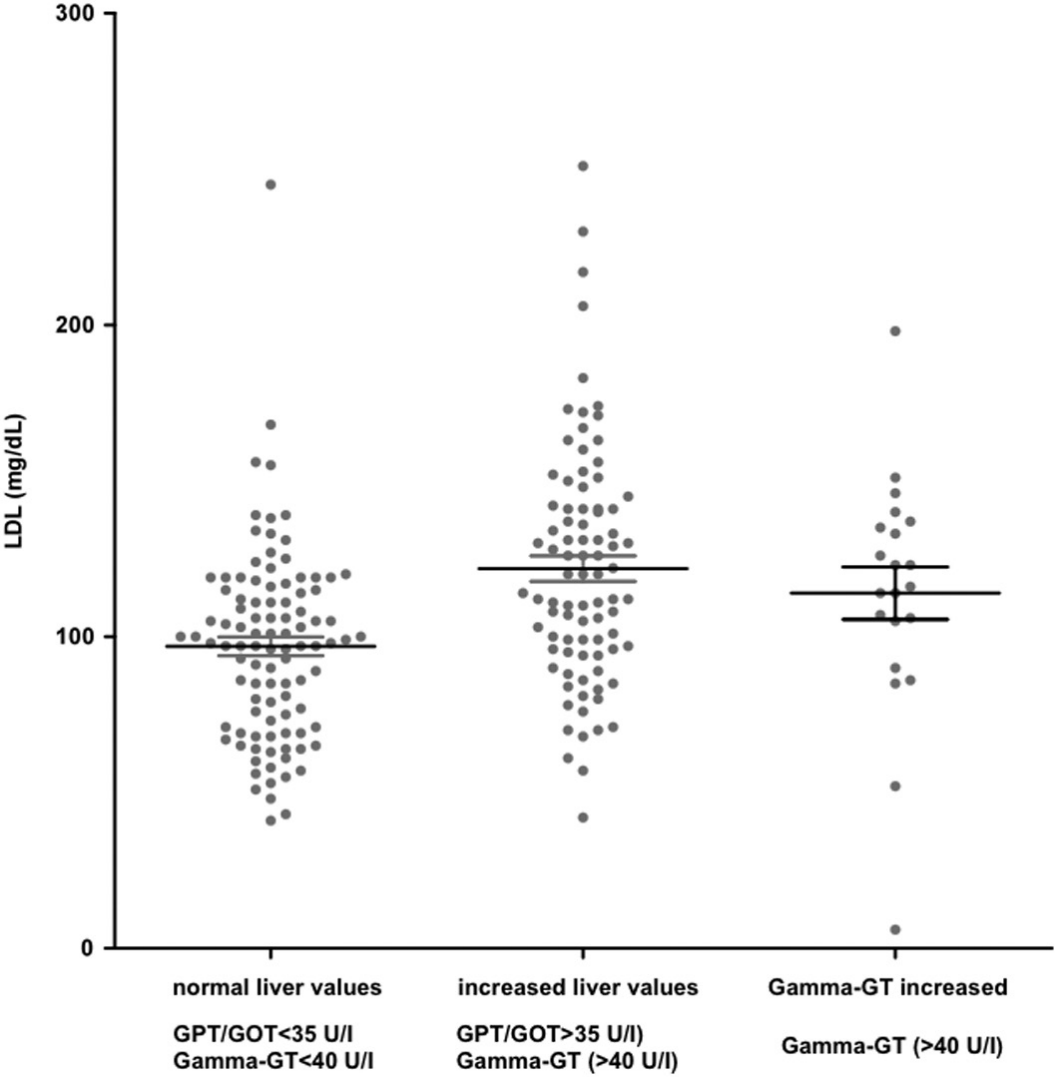
Distribution of low-density lipoprotein (LDL) depending on serum values of liver function. Groups were categorized by: normal liver values (GPT (glutamate pyruvate transaminase)/GOT (glutamate oxaloacetate transaminase) <35 U/I, Gamma-GT <40 U/I), increased liver enzymes (GPT/GOT >35 U/I) and increased Gamma-GT (>40 U/I) (mean ± s.d., *n* = 213).

**Figure 3:**
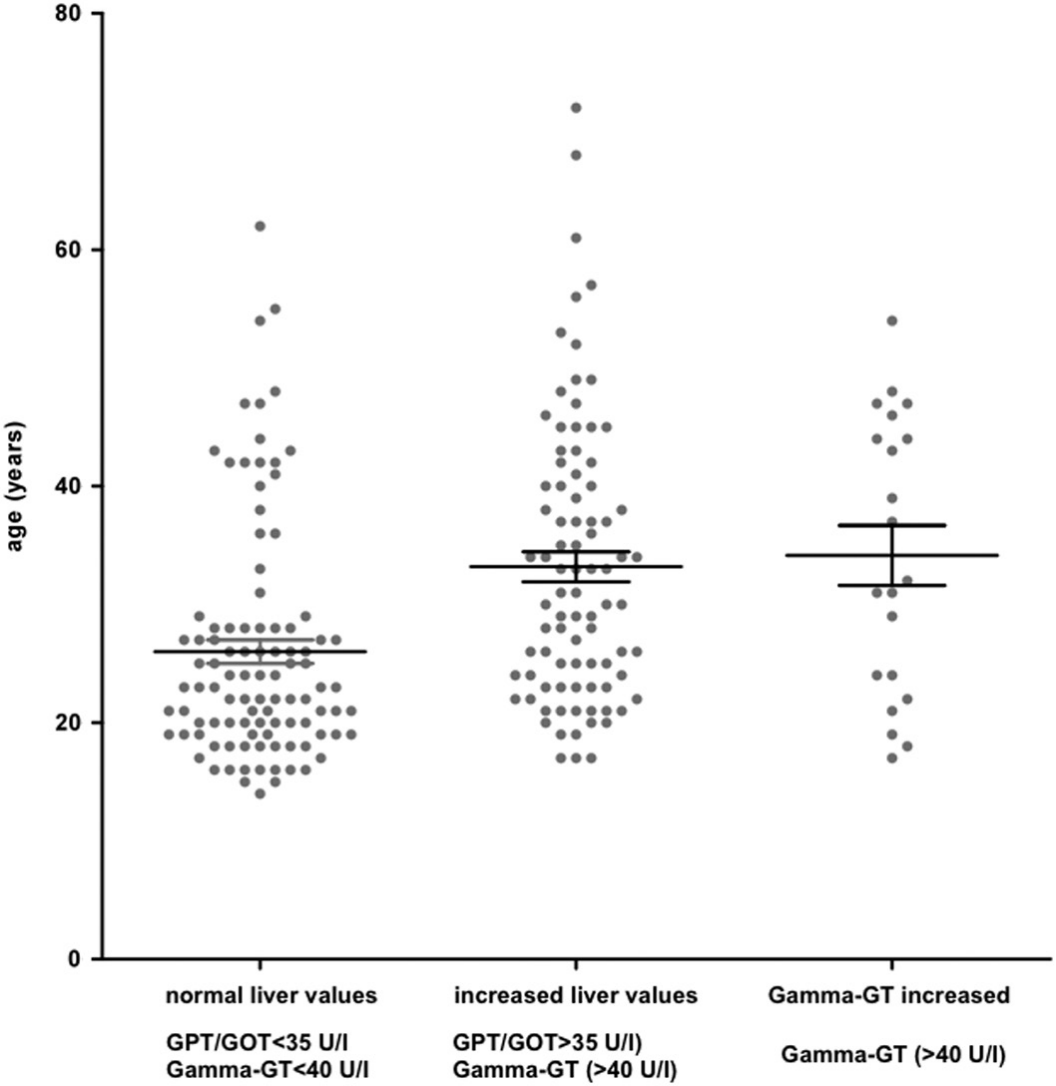
Distribution and comparison of age depending on serum levels of liver function. Groups were categorized by normal liver values (GPT (glutamate pyruvate transaminase)/GOT (glutamate oxaloacetate transaminase) <35 U/I and Gamma-GT <40 U/I), increased liver enzymes (GPT/GOT >35 U/I) and increased Gamma-GT (>40 U/I) (mean ± s.d., *n* = 213).

Given the large number of patients with elevated liver values, we intended to identify influencing factors. We did not find any effect of BMI, HbA1c, or of the type of HRT applied (dermal or oral). In contrast, increasing age and higher LDL levels in women with TS correlated significantly with increased liver values (age: *P* < 0.0001 LDL: <0.0001, multinomial regression) (Figs 2 and 3).

Treatment with hGH was documented for 130 women (50%). Therapy was started at an average age of 9.8 ± 3.4 years and continued for 6.1 ± 3.0 years. We discovered that the start of hGHT correlated significantly with the year of birth. On average, women with a later year of birth started hGHT earlier (regressions coefficient: −0.194, *r*^2^: 0.067, *P* = 0.011; Supplementary Fig. A, see section on supplementary data given at the end of this article). For example, patients born 5 years later started on therapy almost 1 year earlier (0.97 years). The year of birth explained 6% of the variation concerning patient age at onset of hGHT. On average 2 years (2.0 ± 2.8 years) passed between diagnosis and initiation of hGHT. In some cases, Turner syndrome was diagnosed late (6 years and older, *n* = 29), but hGHT was still started with a delay of 1 year in 40% of patients (Supplementary Fig. B).

## Discussion

The medical care of women with TS has been inadequate in many aspects in a number of European countries and still is in Germany. During childhood, patients with TS are often embedded in interdisciplinary pediatric teams that provide medical care. However, during adolescence, around the time of transition, the quality of the medical care provided was seen to worsen, resulting in the founding of coordinated care in different countries and in the development of new guidelines to maintain the level of the medical care provided at an optimum (8, 20, 22, 29, 30, 31, 34, 35).

In Germany, it is estimated that around 16,000 women are currently affected by TS. In this study, we present non-university data from German endocrine centers (Endokrinologikum) before the founding of the Turner-Syndrome Network. Hence, our experience (*n* = 258, 1.6% of all German TS patients) revealed that the TS patients were mainly cared for by their primary care physician and only sent to an endocrinology clinic to control thyroid function, lipid values, and the development of insulin resistance or diabetes.

Looking to a number of other countries, an increase in the morbidity and a deficit in care was detected years ago already (8, 11, 16, 24). Besides the numerous studies from Denmark, the care of TS women was also analyzed in the Netherlands as early as 2007, which resulted in the establishment of a multidisciplinary outpatient team including an internist-endocrinologist, a gynecologist, and a cardiologist (34). The Dutch also identified significant morbidity on analysis of their patients. The authors demonstrated that girls with TS benefit from a careful transition to ongoing adult medical care (29, 34). In Australia, coordinated care also increased the detection of comorbidities with evaluation at a dedicated adult Turner syndrome clinic (22). The Czech Republic announced the necessity of interdisciplinary clinics in 2001 (36), but there are no follow-up data available. In France, a centralized systematic multidisciplinary approach to patients with Turner syndrome from childhood and adolescence to adulthood was also introduced and is regarded as successful (31). In their work, they also suggest the use of a transition readiness assessment provided by The Endocrine Society. This tool comprises different questions concerning health, using healthcare and social and emotional factors. Furthermore, other tests and summary tools, transfer records, and a recommended approach for transitioning into adult practice are available on the Endocrine Society website (http://www.endocrinetransitions.org). Hence, multidisciplinary centers have already been established in the Netherlands, UK, Sweden, and in France, resulting in a better quality of the care available to women with TS including organized transition (26, 29, 31, 32, 35). Austria, like Germany, is far behind these developments (2). Although a working group on TS was initiated in Germany years ago (10, 37), the idea was not successfully taken on board by the endocrinologic community, in direct contrast to the activity in other European countries. We can only speculate as to why this initiative in Germany back in 2013/2014 did not receive sufficient attention to result in the founding of Turner clinics. Within the prevailing non-centralized healthcare system, any initiative is dependent on the degree of personal commitment individual physicians can afford. This is very different from any general decision made by governing bodies of the healthcare system or of a corresponding university to support the foundation of such multidisciplinary centers including organized transition. In addition, endocrinologic departments already have a number of diseases to care for and only limited capacity without additional financial or personal support, which might well have contributed to the present situation.

On the basis of the data underlying this study, we can confirm this deficit in the coordinative care of women with TS. We were interested not only in the quality of endocrine care provided in the five centers studied, but also in the documentation of the involvement of other comorbidities.

The karyotype was documented in the adult patients’ medical records in only 50% of cases. We assumed that the karyotype had been determined previously to confirm the diagnosis in all patients during childhood; however, this specific documentation was missing. Karyotype 45,X (33%) was the most frequent in our patients. Mosaic forms were found in 16%; in nearly 10% parts of the Y chromosome were present. In other studies, karyotype 45,X occurred more frequently (40–50%) than in our data (10, 20, 38). In contrast, the number of our patients in whom parts of the Y chromosome were present was comparable to the literature (3). The difference may be explained by the fact that the exact karyotype was only known in 50% of our women; hence, the real distribution may differ. The distribution of karyotypes in our study may be important, owing to the influence on comorbidities and therefore our results. In contrast to our study, other studies often used the karyotype as inclusion criterion. However, we included patients independent of the knowledge of karyotype present. Given the high risk of developing gonadoblastoma (39, 40) and a number of other comorbidities depending on karyotype (3), the corresponding information is of major importance to the attending physician and should be determined if not already known (33). This has now been included in the routine work-up of patients with TS in these five endocrine centers in Germany.

Spontaneous aortic dissection is one of the main causes of increased mortality in women with TS (18). Cardiac involvement was revealed in only 28% of our patients. This number was very low compared to the published data (41, 42) probably due to inadequate diagnostic work-up or missing documentation in the adult patients’ medical records. The most common heart defects among the 28% were stenosis of the aortic isthmus and a bicuspid aortic valve, as described in the literature (43). Echocardiography was performed in 42% and cardiac MRI in only 8.75%. In a French study, echocardiography was performed on only 21% of adult women with TS if no prior heart defect was known. MRI was not included in the latter study (20). This low number of MRIs performed in our study is particularly alarming, as the importance of cardiac MRI has already been described in a number of studies over several years (41, 42, 44).

One of the main endocrine aspects of women with TS is the thyroid gland. Autoimmune thyroiditis affected 37% of the participants. TPO-AB/MAK-AB was documented in 90% of the patients and in 30% as positive. TRAK-AB was determined in 79% of the women and antibodies were positive in 5%. Substitution with l-thyroxine was necessary in 38.3% of the participants to treat hypothyroidism. In a Danish study, 45% of the patients, with a mean age of 36.7 years, were revealed as positive for TPO-AB; hypothyroidism was found in 33%. There was significant correlation between the presence of antibodies and age (17). Our women were about 7 years younger, possibly explaining the 15% lower prevalence of TPO-AB in our group. However, in another study with younger participants than our group (mean age 26 years), 41% of the TS patients were revealed as positive for TPO-AB. Furthermore, hypothyroidism was found in only 16% in that group. The presence of TPO-AB correlated significantly with the karyotype in which an isochromosome was present. This is a result of a misdivision of the chromosomal centromeres (45). Hypothyroidism was found more frequently in our study than in the literature. It has to be taken into account that our study also included other forms of hypothyroidism without the presence of the respective antibodies. Our results demonstrate that regular screening of the thyroid values and ultrasound examination is required and was performed in the five endocrinologic centers. Medical documentation with respect to the thyroid status in our patients indicates that the endocrine medical care in this regard in these endocrinologic centers was of a high standard.

Women with TS have an increased risk of developing other autoimmune diseases besides thyroiditis, for example, celiac disease (17, 46, 47). In our cohort, no regular screening for other autoimmune disease was implemented until June 2017. Furthermore, no definite screening is proposed in the guidelines (48). However, it has been suggested lately that HLA testing is to be succeeded by regular antibody testing if positive (49).

The reason for the high prevalence of increased liver parameters remains unclear (14, 50). However, TS predisposes to severe liver disease (51). We detected increased liver parameters in more than 50% of the patients included in this study. When further analyzing influencing factors, we were able to detect a significant correlation with elevated LDL levels and increased age, confirming data from Sweden (52). This prevalence decreased on treatment with HRT (50, 51, 53). Moreover, higher BMI correlated to increased liver values in the Swedish cohort (52). We were nevertheless unable to determine any correlation between BMI and increased liver parameters. The high prevalence of increased liver values underlines the importance of routine testing, including lipid parameters in particular with increasing age. The effect of lipid-lowering therapy would be interesting in this regard and should be addressed in prospective studies. Our data also revealed that hepatic involvement was at least detected in the participating endocrinologic centers. The consideration of risk-adapted treatment options for increased LDL levels in daily patient care might improve the health of women with TS (33).

Treatment with growth hormone to improve adult height in particular is one of the main aspects of such care and has been implemented as a therapeutic measure since 1991. No official recommendations for when to initiate hGHT existed at the time the patients in our study started on this treatment. However, the age at onset of hGHT has been lowered over the last few years, with the current recommendation (since 2016) suggesting 4–6 years of age (33). Therefore, the late onset at an average age of 9.8 years in our study, which presents data from 2001 to 2017, reflects the developing indication for early growth hormone therapy in Germany during this period. Retrospectively, the late onset can be interpreted as insufficient care of TS patients with respect to growth therapy.

### Limitations of the study

As a retrospective study, the data were incomplete, despite the relatively high number of patients. Patients were seen in these centers mostly only for the care of endocrine problems. Therefore, only limited data are available. The exact values of liver enzymes and thyroid antibodies were not documented as numbers, only categorized as either increased or normal. We only included TS patients from these five endocrinologic centers in our study. As such, our findings may not be applied to the care of TS women in Germany in general.

Our study covering data until June 2017 demonstrates that the endocrine diagnostics of the thyroid and metabolic status of women with TS in the endocrinologic centers was available in 90% of the patients. However, investigations recommended by the guidelines from 2017 were documented only in a small number of the women in our retrospective study. The medical care with respect to not only cardiovascular involvement but also other autoimmune disease underlines the need for improvement. In the participating centers, a structured work-up according to the guidelines is now implemented to improve the care of TS women (Supplementary Table 1).

The complex health problems that women with TS present require responsible interdisciplinary cooperation and can be optimized in specialized centers, as already established in several European countries and in the United States. Meanwhile, the Turner-Syndrome Network was founded during the German Congress of Endocrinology in Göttingen, Germany, in March 2019. This successful development was the result of a working-group consisting of the German Turner-Syndrome Association (patient support group), the German Society of Pediatric Endocrinology and Diabetology, and the German Endocrine Society. The clinicians interested and experienced in the care of individuals with Turner Syndrome and fulfilling certain criteria were invited to apply to be part of the network as Turner specialists or Turner Centers. In order to guarantee an optimized transition process from pediatric to adult care, the application needed to include both a pediatric and an adult endocrinologist in combination.

The following years will reveal whether, after structured transition, the attending adult endocrinologist can be the coordinator for TS women, responsible for initiating the necessary investigations and collecting all the relevant medical information, including that concerning non-endocrine involvement. We hope that the data will be available at some point in the future following this development.

## Supplementary Material

supplementary Figure A

supplementary Figure B

## Declaration of interest

The authors declare that there is no conflict of interest that could be perceived as prejudicing the impartiality of the research reported.

## Funding

This research did not receive any specific grant from any funding agency in the public, commercial or not-for-profit sector.
