# Supplementary material for: Deficient knowledge in adult Turner syndrome care as an incentive to found Turner centers in Germany
Source: Endocr Connect. 2019 Oct 18;8(11):1483–92. doi: 10.1530/EC-19-0418 (PMC6865863; doi:10.1530/EC-19-0418)
Supplement: supplementary Figure A [file supplementary_figure_1.pdf]

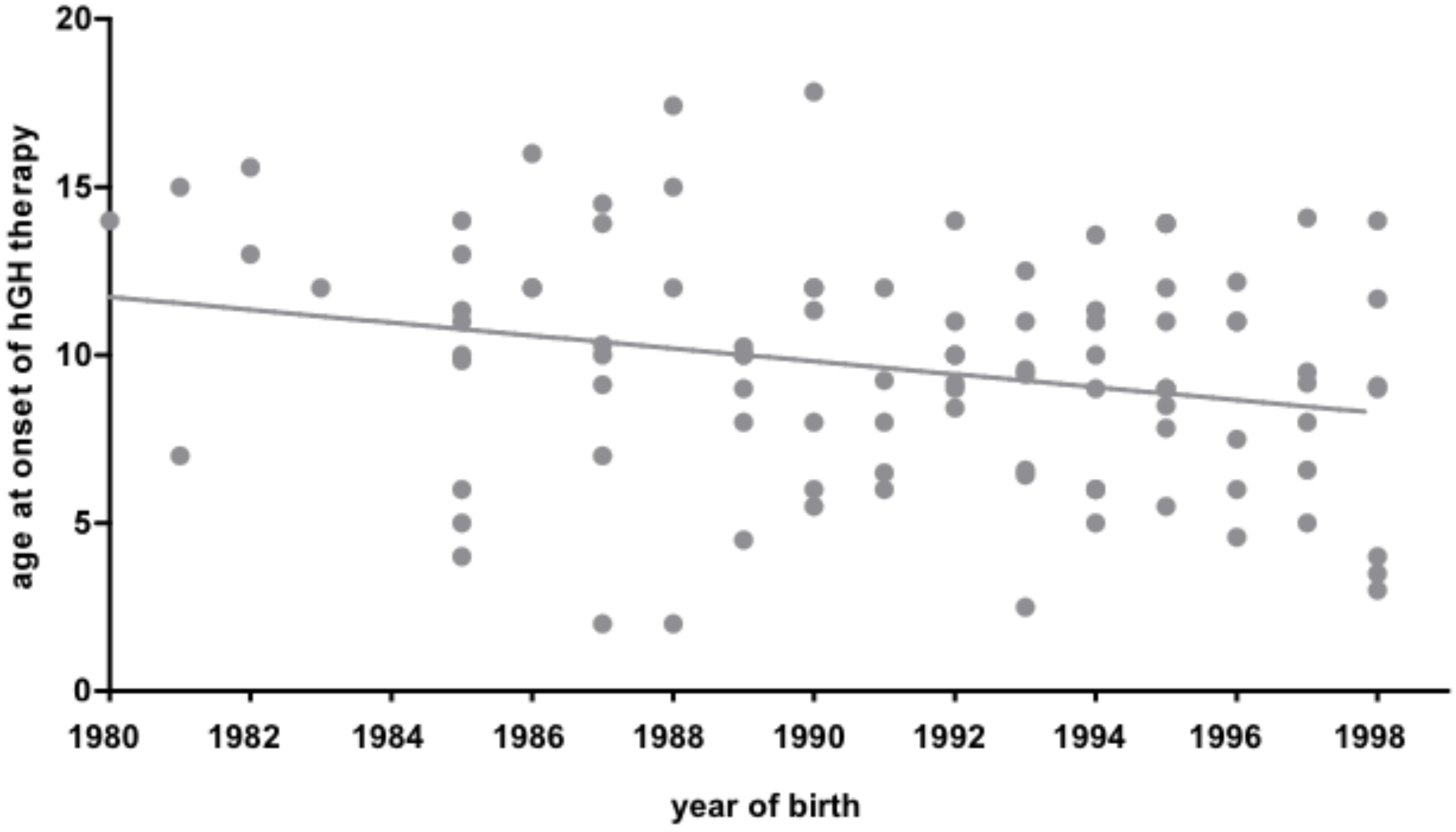

Supplementary Figure A: Effect of the year of birth (1980 to 1998) on the age at onset of hGH therapy (in years) N=97; The linear regression line is defined by y= - 0.194x+11.73
