# Supplementary material for: Deficient knowledge in adult Turner syndrome care as an incentive to found Turner centers in Germany
Source: Endocr Connect. 2019 Oct 18;8(11):1483–92. doi: 10.1530/EC-19-0418 (PMC6865863; doi:10.1530/EC-19-0418)
Supplement: supplementary Figure B [file supplementary_figure_2.pdf]

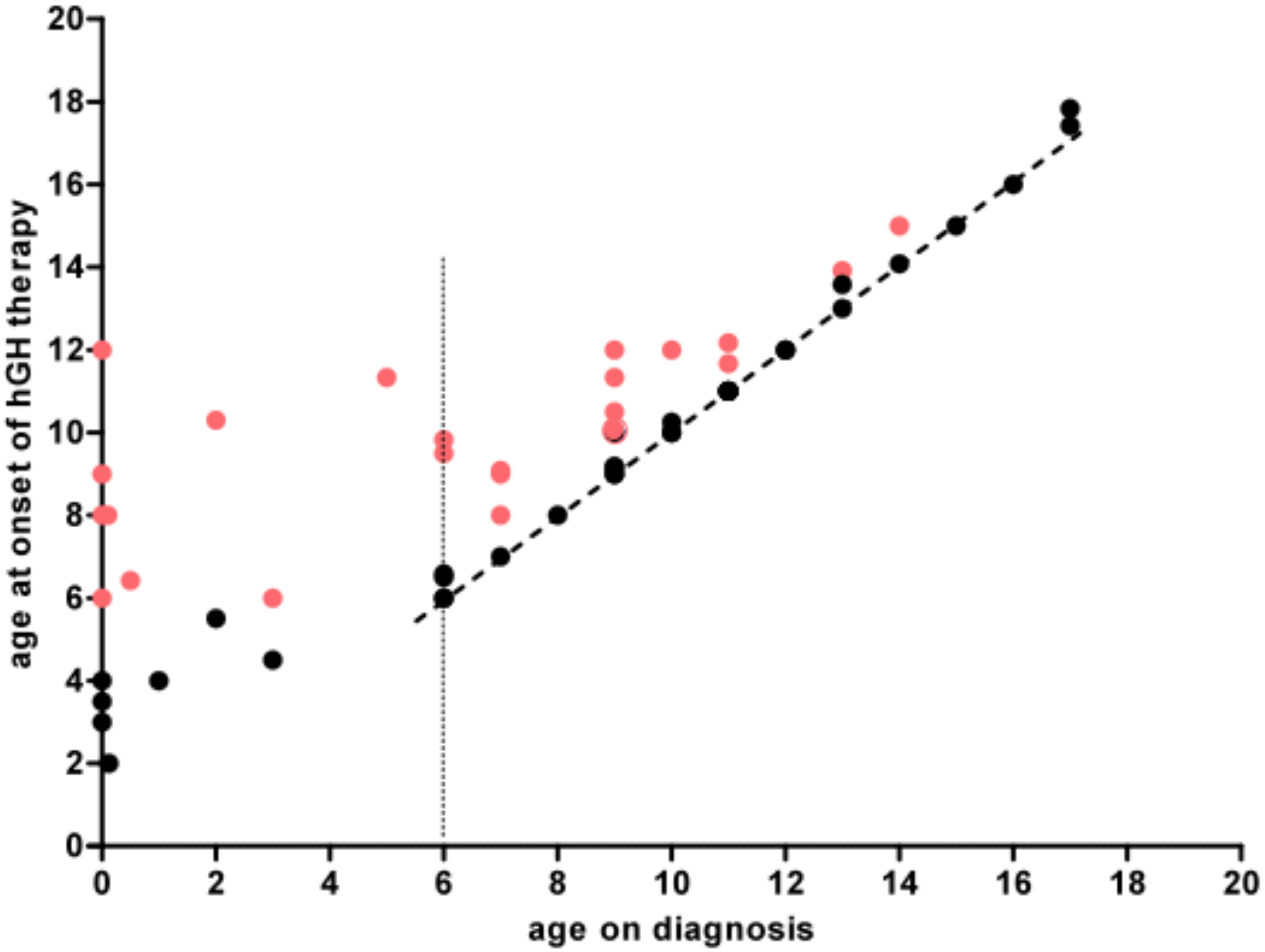

Supplementary Figure B: Relationship between the age on diagnosis and the age at onset of hGH therapy for TS girls in this study (n=58). The diagonal line indicates those women diagnosed at the greater ages of six to 18 years and whose hGH therapy began within the same year. Red data points on the right hand side indicate patients (age on diagnosis ≥ six years) who received hGH between one and up to four years following diagnosis. The patient group with an age on diagnosis < six years is depicted on the left hand side of the diagram. Black data points indicate girls receiving hGH in a time window corresponding to the current guidelines. The red data points indicate those girls who received hGH between the ages of six and older (up to 12 years of age), although they were diagnosed three to 12 years earlier.
